# Morbidity and Mortality of Typhoid Intestinal Perforation Among Children in Sub-Saharan Africa 1995–2019: A Scoping Review

**DOI:** 10.1007/s00268-020-05567-2

**Published:** 2020-05-19

**Authors:** Megan Birkhold, Yacaria Coulibaly, Oumar Coulibaly, Philadelphie Dembélé, Daniel S. Kim, Samba Sow, Kathleen M. Neuzil

**Affiliations:** 1 Department of Surgery, University of Maryland School of Medicine, 22 S. Greene St, S8B02, Baltimore, MD 21201 USA; 2 Service de Chirurgie pédiatrique, CHU Gabriel Touré, Bamako, Mali; 3 Department of Surgery, Koutiala Women’s and Children’s Hospital, Koutiala, Mali; 4 Center for Vaccine Development - Mali, Bamako, Mali; 5 Center for Vaccine Development and Global Health, University of Maryland School of Medicine, Baltimore, MD USA

## Abstract

**Background:**

Typhoid fever incidence and complications, including intestinal perforation, have declined significantly in high-income countries, with mortality rates <1%. However, an estimated 10.9 million cases still occur annually, most in low- and middle-income countries. With the availability of a new typhoid conjugate vaccine licensed for children and recommended by the World Health Organization, understanding severe complications, including associated mortality rates, is essential to inform country-level decisions on introduction of this vaccine. This scoping review summarizes over 20 years of the literature on typhoid intestinal perforation in sub-Saharan Africa.

**Methods:**

We searched EMBASE, PubMed, Medline, and Cochrane databases for studies reporting mortality rates due to typhoid intestinal perforation in children, under 18 years old, in sub-Saharan Africa published from January 1995 through June 2019.

**Results:**

Twenty-four papers from six countries were included. Reported mortality rates ranged from 4.6–75%, with 16 of the 24 studies between 11 and 30%. Thirteen papers included postoperative morbidity rates, ranging from 16–100%. The most documented complications included surgical site infections, intra-abdominal abscesses, and enterocutaneous fistulas. High mortality rates can be attributed to late presentation to tertiary centers, sepsis and electrolyte abnormalities requiring preoperative resuscitation, prolonged perforation-to-surgery interval, and lack of access to critical care or an intensive care unit postoperatively.

**Conclusions:**

Current estimates of mortality related to typhoid intestinal perforation among children in sub-Saharan Africa remain unacceptably high. Prevention of typhoid fever is essential to reduce mortality, with the ultimate goal of a comprehensive approach that utilizes vaccination, improvements in water, sanitation, and hygiene, and greater access to surgical care.

**Electronic supplementary material:**

The online version of this article (10.1007/s00268-020-05567-2) contains supplementary material, which is available to authorized users.

## Introduction

Typhoid fever is caused by the ingestion of contaminated food or water with the bacterium *Salmonella enterica* serovar Typhi (*S*. Typhi), from either an acutely infected person or a chronic carrier [1]. Children are disproportionately affected with increased incidence seen between 5 and 14 years of age [2]. Over the past century, public health efforts, including improved sanitation infrastructure, access to clean water, and availability of effective antibiotics, have contributed to the decline of typhoid fever incidence and complications, including intestinal perforation, in high-income countries [2, 3]. In the USA, there were an estimated 350 cases of typhoid fever annually from 2008 to 2015, with an associated mortality rate of less than 1% [4]. The majority of these cases were due to travelers from endemic areas [1]. In comparison, typhoid fever continues to be a significant contributor to morbidity and mortality in low- and middle-income countries (LMICs) in sub-Saharan Africa and southeast Asia, especially in pediatric populations, where incidence peaks in children five to nine years of age [5].

The 2019 Global Burden of Diseases, Injuries, and Risk Factors study estimated that 10.9 million cases of typhoid fever occurred worldwide in 2017, with about 116,800 attributable deaths [5]. An estimated 12.1% of the global cases of typhoid fever occurred in sub-Saharan Africa [5]; however due to limitations with typhoid diagnostic data and modeling, as well as generalizability across the continent, this number is likely an underestimate of the true burden of disease for this region.

A feared complication of typhoid fever is typhoid intestinal perforation (TIP), observed in an estimated 0.8% to 39% of untreated or improperly treated cases worldwide [6]. This is especially true in medically resource poor areas, including sub-Saharan Africa, which often lack access to specialized surgical and postoperative care. In addition, TIP occurs more often in children, with increased mortality rates when compared to adults [7]. In the 1960s and 1970s, TIP-associated mortality rates in all ages ranged from around 30% in Ghana [8] and India [9] to about 60% in Nigeria [6]; a reported rate of 30.5% was seen in a study of Guatemalan children [10]. Since 1990, mortality rates in some endemic areas have decreased to between 3 to 13% in Pakistan [11, 12], Nepal [13], and Iraq [14], with a study from Turkey reporting a rate of 4.8% in children [15]. Data specific to TIP in children living in sub-Saharan Africa are limited.

In 2018, the World Health Organization (WHO) recommended the use of a new typhoid conjugate vaccine (TCV) to prevent typhoid fever and its complications among children age six months and older in endemic areas [2]. Other currently available vaccines have limited duration of protection and are only recommended for children two years of age and older, making it difficult to incorporate these vaccines into the routine immunization schedule [16]. Importantly, Gavi, the Vaccine Alliance (Gavi), committed to co-finance the TCV in eligible countries, the majority of which are located in sub-Saharan Africa [17, 18]. In order to assess the full impact of this vaccine and help inform country decisions on introduction, understanding severe complications is essential. The last mortality-focused review of TIP worldwide was published in 1994 and included all ages [19]. As a review of TIP specific to children living in sub-Saharan Africa has not been done, current mortality rates are unknown. For these reasons, we conducted a scoping review to obtain mortality rates due to TIP in children, under 18 years of age, living in sub-Saharan Africa, in order to summarize and update the field and to identify gaps in current knowledge.

## Methods

This review was conducted utilizing the methodology from Arksey and O’Malley’s framework for scoping studies [20]. Our primary research question was to identify the currently available literature on mortality rates of typhoid intestinal perforation in children, under 18 years of age, living in sub-Saharan Africa. We also reviewed reported morbidity rates and surgical complications when available.

Our search strategy was developed in consultation with typhoid fever experts, pediatric surgeons from the sub-Saharan African region, and a university librarian. We intentionally used broad search terms and did not limit by age, country, or language, in order to optimize the search. Key teams included typhoid, typhoid fever, enteric fever, perforation, peritonitis, and ileal. We searched EMBASE, Medline, PubMed, and Cochrane Library electronic databases for articles, abstracts, and reviews published from January 1, 1995, to June 30, 2019 (Online Resource 1). Case reports were excluded as they generally only report on one or two patients, thus artificially inflating or deflating associated mortality rates.

All titles and abstracts identified in the search were screened for the following inclusion criteria: publication date from January 1, 1995 to June 30, 2019, study participants younger than 18 years of age, study site in sub-Saharan Africa, and mortality rate due to TIP reported or able to be calculated from the raw data provided. Studies of surgical abdominal emergencies or generalized peritonitis were included if they reported data specific to TIP separately and met all other criteria. Studies not meeting these criteria were excluded. We reviewed full text articles that appeared to fit the inclusion criteria.

For each included study, data were extracted and entered into a Microsoft Excel sheet that included author names, date of publication, study location, study design, level of hospital, study period, number of patients, age range, mortality rate, postoperative morbidity rate, surgical complications, and culture and pathology data. A limited statistical analysis was performed using Microsoft Excel and SAS version 9.4. A descriptive analysis was performed to summarize the findings and help guide future research.

## Results

**Fig. 1:**
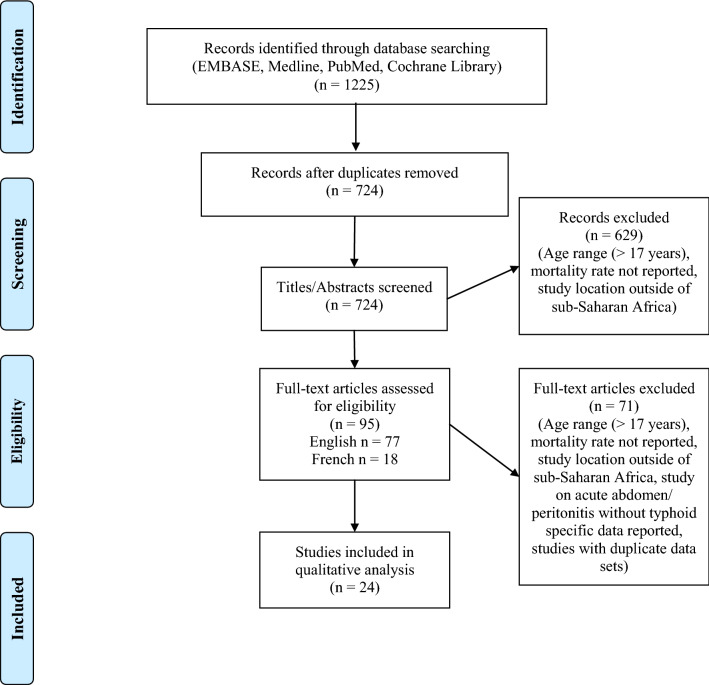
Flow diagram of studies screened and included in the final review

**Fig. 2:**
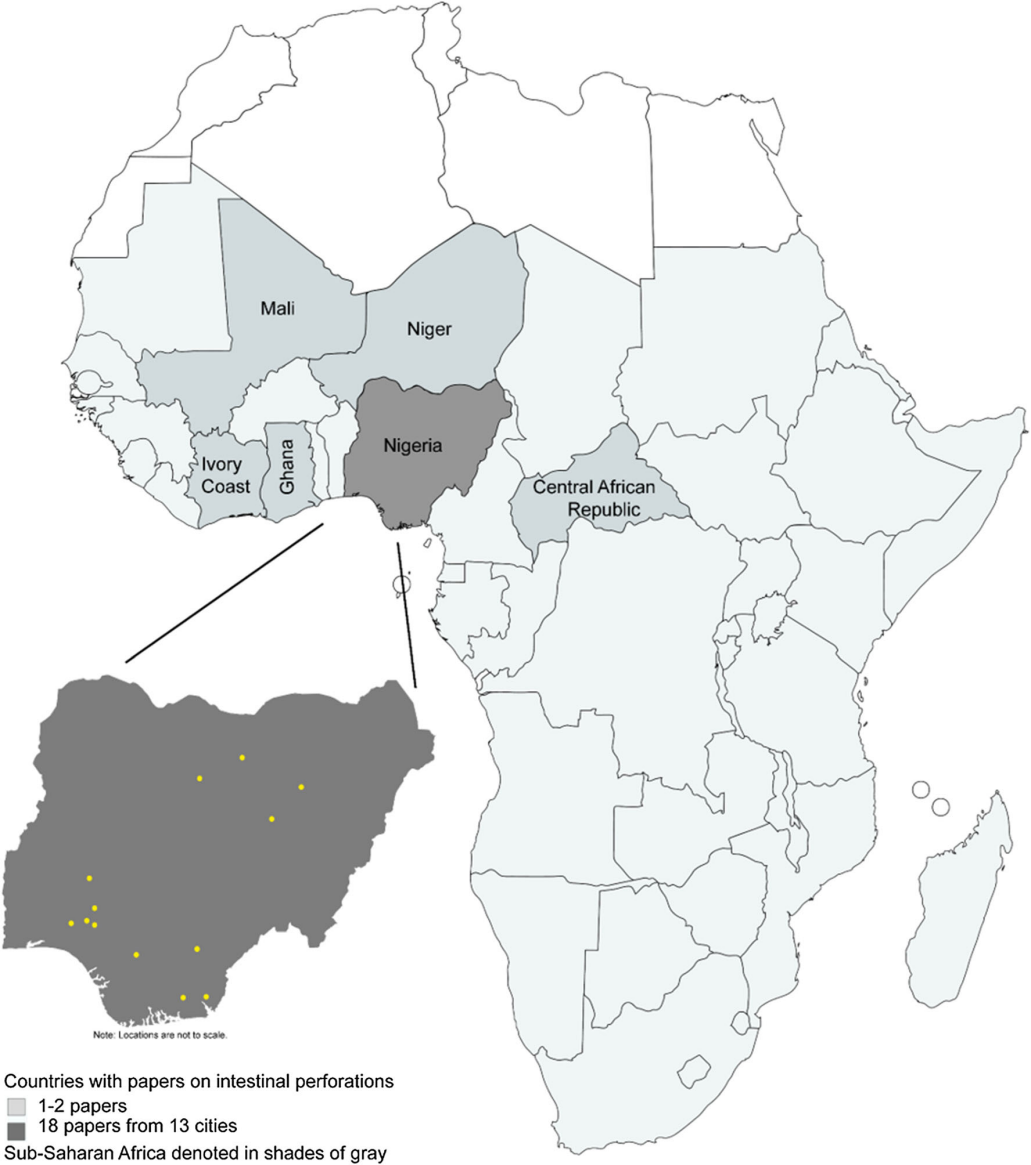
Map of Africa showing the distribution of included papers

A total of 1,225 articles were identified during the search (Fig. 1). After removal of duplicates, 724 titles and abstracts were screened. The second screening included 95 full text articles in English and French. Of the 27 identified papers that met the inclusion criteria, three papers were excluded because they were sub-studies of papers using the same data that were already included. The final review included 24 papers [21–44] with studies conducted from 1984 to 2018, of which 20 were retrospective and four prospective studies. Eighteen of the studies were conducted in Nigeria, two from Ghana, and one each from the Central African Republic, Ivory Coast, Mali, and Niger (Fig. 2). All of the studies were from tertiary healthcare centers. Total numbers of patients included in each study ranged from 9 to 902. Table 1 summarizes study details including location, study years, number of patients, age of patients, postoperative morbidity rate, and mortality rate.

**Table 1:** Characteristics of the 24 included studies with associated morbidity and mortality rates

Authors	Place of study	Study years	Number of patients	Age range (years)	Postoperative morbidity^a^ n (%)	Mortality *n* (%)
*Studies from Africa (excluding Nigeria)*						
Kouame et al. [37]	Abidjan, Ivory Coast	1990–2000	48	3–16	22 (46)	3 (6)
Abantanga et al. [25]	Kumasi, Ghana	1995–1997	121	4–14	60 (49.5)	15 (12.4)
Bobossi Séréngbé et al. [31]^b^	Bangui, Central African Republic	1997–1998	31	10 months–15	5 (16)	9 (29)
Abantanga et al. [27]	Kumasi, Ghana	2001–2005	650	1–14	241 (37.1)	82 (12.6)
Coulibaly et al. [26]	Bamako, Mali	2005–2010	105	3–14		16 (15.2)
Adamou et al. [30]	Zinder, Niger	2013–2015	153	< 15		22 (14.4)
*Studies from Nigeria*						
Rahman et al. [39]	Ilorin, Nigeria	1984–1999	106	3–14	56 (53)	25 (23.6)
Irabor [32]	Ibadan, Nigeria	1985–2000	183	3–14		39 (21.3)
Ameh [40]	Zaria, Nigeria	1987–1996	64	2 months–12	34 (53)	25 (39)
Osifo et al. [41]	Benin City, Nigeria	1993–2007	12	5–13	12 (100)	9 (75)
Usang et al. [28]	Ile-Ife, Nigeria	1994–2004	38	1–15		9 (23.7)^d^
Ekenze et al. [38]	Enugu, Nigeria	1995–2004	89	1–15	50 (56.9)	17 (19.1)
Uba et al. [33]	Jos, Nigeria	1996–2005	184	4–15	98 (53.3)	42 (22.8)
Ekenze et al. [22]	Enugu, Nigeria	2001–2006	83	9 months–15	49 (59)	21 (25.3)
Nasir et al. [24]	Ilorin, Nigeria	2002–2009	153	3–15		16 (10.4)
Nuhu et al. [29]	Azare, Nigeria	2004–2008	46	1–15		13 (28.3)
Talabi et al. [44]	Ife, Nigeria	2005–2013	45	2–15	31 (86.1)^c^	9 (20)
Usang et al. [21]	Calabar, Nigeria	2006–2015	49	5–15		4 (8.2)
Ibrahim et al. [42]	Kano, Nigeria	2007–2012	902	3–14	469 (52)^d^	42 (4.6)
Ekenze et al. [43]^b^	Enugu, Nigeria	2008–2009	22	2–15	15 (68.2)	3 (13.6)
Adegoke et al. [23]	Ado-Ekiti, Nigeria	2008–2010	47	2–15		6 (12.8)
Anyanwu et al. [34]	Kano, Nigeria	2009–2013	129	3–13		14 (10.9)
Ajao et al. [36]^b^	Ibadan, Nigeria	2010–2017	9	< 15		1 (11.1)^d^
Ekpemo et al. [35]	Aba, Nigeria	2016–2018	60	3–15		5 (8.3)

^a^Not all papers reported postoperative morbidity; those cells were left empty

^b^Papers included on acute abdominal emergencies or generalized peritonitis with typhoid intestinal perforation specific data available

^c^Morbidity percentage reported only for survivors (*n* = 36)

^d^Percentage not reported by authors; raw data provided in the paper were used to calculate the percentage

The mortality rate across studies ranged from 4.6 to 75%, with 16 of the 24 between 11 and 30% (Table 1). An overall mortality trend was unable to be calculated due to the nature of the reported data and overlap of study years. A percent change in mortality calculation was performed for four cities in Nigeria, as each city contained two studies without overlapped time periods [24, 28, 32, 36, 38, 39, 43, 44]. Each of these cities showed a declining mortality trend overall, with a percent change of −28.8% (decrease in mortality rate from 19.1 to 13.6%) in Enugu, −47.9% (21.3 to 11.1%) in Ibadan, −15.6% (23.7 to 20%) in Ife, and −56.3% (23.8 to 10.4%) in Ilorin.

Thirteen papers included postoperative morbidity rates, ranging from 16 to 100%. Postoperative complication data were provided in 19 papers [21, 22, 24–26, 28, 29, 32–35, 37–44] (Table 2). Not all papers reported every complication listed in the table. Surgical site infection, intra-abdominal abscess, enterocutaneous fistula (ECF), and wound dehiscence rates were documented by the majority of included studies. Due to the lack of consistent reporting of complications, different lengths of time with significant overlap of years, and non-standardized outcome definitions across the studies, a valid statistical comparison for morbidity trends and a calculation for the overall morbidity rate was unable to be performed.

**Table 2:** Percentage of the eight most common postoperative complications reported^a^

Authors	Number of patients	Surgical site infection (%)	Chest infection^b^ (%)	Intra-abdominal abscess (%)	Enterocutaneous fistula (%)	Wound dehiscence (%)	Evisceration (%)	Incisional hernia (%)	Re-perforation (%)
Kouame et al. [37]	48	16.7			12.5			10.4	
Abantanga et al. [25]^c^	121	48.8	7.4		2.5	20.7	3.3	2.5	
Coulibaly et al. [26]^c^	105	14.3			3.8		4.8	9.5	
Rahman et al. [39]	106	54.0			6.3				
Irabor [32]	183	87.4			15.8	21.9		21.9	
Ameh [40]^d^	64	53.3	30.0	6.7		13.3			6.7
Osifo et al. [41]^c^	12	25.0			41.7		16.7		
Usang et al. [28]	38	53.1		6.3	6.3	15.0			
Ekenze et al. [38]	89	46.1	23.6	2.2		7.9			21.3
Uba et al. [33]	184	89.1	53.3	12.5	24.5	55.4	9.8	2.7	
Ekenze et al. [22]	83	51.8	14.5	6.0		12.0		9.6	7.2
Nasir et al. [24]^c^	153			1.3	7.8	7.8	4.6		
Nuhu et al. [29]	46	45.6	8.7	2.2	8.7	13.0			19.6
Talabi et al. [44]^c^	45	64.4		4.4	8.9	31.1	13.3		2.2
Usang et al. [21]	49	40.8	10.2		10.2		4.1		
Ibrahim et al. [42]	902	33.9			6.6		4.5		
Ekenze et al. [43]^c^	22	45.4				9.1		9.1	4.5
Anyanwu et al. [34]	129	68.2		12.4	10.1	20.9	13.2		
Ekpemo et al. [35]	60	83.3	50.0	50.0	8.3				

^a^Not all complications reported are included in the table, only those reported by most papers; not all papers reported every postoperative complication. In those that did not, cells were left empty

^b^Chest infections include pneumonia and/or empyema

^c^Percentage not reported by paper; calculated with the total number of patients in the study as the denominator

^d^Percentage reported based on survivors only (*n* = 30) not on total number of patients in the study

## Discussion

This current review found that slight progress has been made in reducing TIP-associated mortality over the past 50 years in sub-Saharan Africa. From 1984 to 2018, mortality rates ranged from 4.6 to 75%. In the data available since 2000, 79% of the reported mortality rates were between 10 and 30%. Although these numbers are moving in the right direction, they are still unacceptably high. In a tertiary care hospital in Ghana, the mortality rates for the two included studies did not change over a 10-year period, remaining just over 12% [25, 27]. Due to limited data, only one paper each from the Central African Republic, Ivory Coast, Mali, and Niger was included, making it impossible to determine temporal trends. The cities of Enugu, Ibadan, Ife, and Ilorin in Nigeria each had two studies, without overlapping time periods, allowing for a percent change in mortality calculation [24, 28, 32, 36, 38, 39, 43, 44]. Findings showed a declining mortality trend overall within these cities, with the percent change ranging from a 15.6% decrease in Ife to a 56.3% decrease in Ilorin. This data should be interpreted with caution, however, given the wide ranges of study years these mortality rates represent, some including data from over 10 years, and the varying number of study participants. In the Ibadan, Nigeria study from Irabor [32], the overall mortality rate for the 15-year study period was 21.3%. However, when separated into five-year intervals, the mortality rate increased from 15.8 to 30.4% during the entire study period. Consistent reporting of yearly mortality rates by authors would allow a more accurate calculation and interpretation of overall mortality rate trends for this region.

The continued high mortality rates seen in many studies were attributed to several factors in the perioperative period. Late presentation by patients due to a delay in diagnosis, as well as difficulty accessing adequate medical care in rural areas, was common [43]. As all the hospitals included in this review were tertiary care centers, many of the patients had presented to outpatient clinics or traditional healers prior to admission. Many patients had sepsis and electrolyte abnormalities on admission requiring preoperative resuscitation. These factors led to a prolonged perforation-to-surgery interval, which has been shown to increase morbidity and mortality [29, 38, 41, 44]. In addition, the number of perforations present, lack of access to critical care management or an intensive care unit, and inadequate culture data to guide antibiotic therapy contribute to poor outcomes [7, 35].

In addition to mortality, postoperative morbidity remains unacceptably high in LMICs. As shown in Table 1, there was a wide range, 16% to 100%, reported in the included studies, with 62% of the rates falling between 40 and 60%. All studies from Nigeria had a morbidity rate above 50%. Surgical site infection was the most reported complication followed by ECF formation and wound dehiscence. Similar to the high mortality observed with TIP, these complications are likely a result of the late presentation of patients, inadequate or inappropriate antimicrobial therapy, and extensive peritoneal contamination with feculent material leading to dirty wounds. Evisceration was reported in nine studies [21, 24–26, 33, 34, 41, 42, 44] which led to additional surgical procedures and mortality rates ranging from 25 to 100% in the four studies that reported mortality [21, 25, 41, 44].

A dreaded complication of intestinal surgery is the development of an ECF. This is especially true in many LMICs where, due to the lack of resources for management of this problem, including access to total parenteral nutrition, patients are dying from high-output fistulas. Fifteen studies [21, 24–26, 28, 29, 32–35, 37, 39, 41, 42, 44] reported this as a complication with a mortality rate ranging from 40% in one study [21] where five ECFs were seen to 75% in two studies [26, 41] with four patients each. High postoperative morbidity rates lead to increased hospital length of stay, which in turn causes an undue financial strain on families who have to pay for medical bills and stay at the hospital to help provide postoperative care for their loved ones [44, 45].

In order to reduce the mortality and postoperative morbidity associated with TIP, a multifactorial approach is necessary. The prevention of typhoid fever with routine immunization of children with TCV, in conjunction with improved sanitation infrastructure and robust community education on health and proper hygiene practices, is ideal [21, 22, 27, 29, 31, 32, 34, 35, 37–41, 43]. However, once a child has typhoid, proper interventions to stop disease progression are lifesaving. Many authors reported on the need for a higher suspicion of typhoid when a child presents with a fever, as often they are first diagnosed with and treated for malaria [21–23, 26, 29, 31, 39, 43]. This assumption of malaria delays proper antibiotic treatment and allows progression of the disease, which can lead to TIP. Additionally, educating caretakers on the signs and symptoms of typhoid fever, its complications, and when to go to a health center or hospital are essential to ensure children are presenting early in the disease course, not weeks after onset [21, 22, 31, 40, 44]. If TIP does occur, prompt recognition will help decrease the perforation-to-surgery time internal, an important factor which could have a considerable impact on morbidity and mortality [22, 23, 25, 29, 31, 33, 35, 36, 38, 39, 41, 44].

**Fig. 3:**
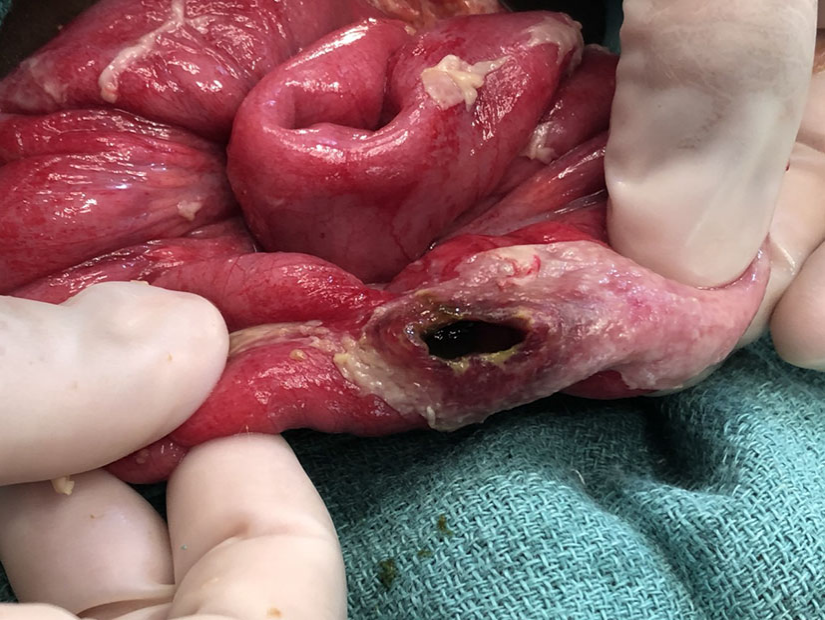
Intra-operative photograph showing a typhoid intestinal perforation lesion of the terminal ileum

Many publications in this review relied on clinical aspects to diagnose TIP. Perforations due to typhoid usually occur in the terminal ileum and are widely thought to occur due to necrosis of Peyer’s patches, leading to ulceration and perforation. This produces a recognizable lesion of an oval perforation located longitudinally along the antimesenteric border (Fig. 3) [46]. When seen in conjunction with clinical signs for typhoid fever, this lesion is normally considered pathognomonic; however, other causes of non-traumatic bowel perforation can be hard to rule out, especially when culture data are unavailable. For example, cytomegalovirus (CMV) enteritis and intestinal tuberculosis (TB), which can present similarly to typhoid fever, can be rare causes of perforation mostly seen in the colon, duodenum, and ileum [47–50]. Intestinal TB more often presents with ulcerations, with the long axis located perpendicular to the long axis of the bowel [49, 50], unlike in TIP. CMV enteritis is usually seen in immunocompromised patients, especially in conjunction with HIV, and has no associated seasonal variation [47, 48, 51]. In contrast, typhoid has been shown to have a seasonal incidence, and five of the papers showed correlations between TIP and the usual typhoid season in their region [27, 32–34, 40].

A definitive etiologic diagnosis was uncommon in the papers reviewed. This was due to both underutilization and lack of sensitivity of cultures. Only five studies reported using blood, stool, or peritoneal fluid cultures to aid in definitive diagnosis [22, 23, 31, 37, 38], with just one study having a confirmed positive culture on all included patients [23]. The percentage of *S*. Typhi positive cultures among suspected cases ranged from about 25% to 50% for blood, stool, or peritoneal fluid. Many patients present later in the disease course, with some having already taken an antibiotic, lowering the sensitivity of cultures. As a result, the diagnosis is usually clinical and confirmed during surgery [33].

This review has several limitations and was restricted by the quantity and quality of data from sub-Saharan Africa available in the published literature. First, it is highly likely that studies were missed due to publication in local or regional journals. Second, even without language restrictions, our search only resulted in the inclusion of four non-English studies. It is possible research from this region is published in languages other than English or French and is not readily available to international readers. Third, the data presented are limited to 6 of the 46 countries in sub-Saharan Africa, with 75% from Nigeria. While there were additional papers from other countries with data from adults, studies specific to the pediatric population are lacking. As incidence and severity of TIP are increased in this age group, and as this is the population that will benefit most from the new TCV, having pediatric-specific data is imperative.

Generalizability is difficult both across this region and within individual countries. Postoperative morbidity and mortality rates were available from only one or two studies in five out of the six countries represented. In the case of Ghana, both studies were from the same urban health center, which makes it difficult to extrapolate these data countrywide. Additionally, all the studies were from tertiary healthcare centers, with the majority retrospective in nature. This can lead to referral and selection biases, which again can make it difficult to generalize to surrounding regions and likely underestimate the actual mortality related to TIP. It is highly probable that many children with TIP die before they ever reach a tertiary care center.

With the ongoing high morbidity and mortality rates seen with TIP in sub-Saharan Africa, a multi-system approach is needed for prevention of typhoid fever, as well as to improve health systems and access to quality surgical care. The new WHO recommended single-dose TCV is now available for children over six months of age and was recently shown to be 81.6% efficacious in preventing typhoid fever in a randomized, controlled clinical trial in Nepal [52]. Thus far, only Pakistan, India, and Zimbabwe have begun to use this vaccine. In response to a typhoid outbreak in early 2019, Zimbabwe implemented a mass vaccine campaign which resulted in a decrease in typhoid cases seen among children [53]. In addition, Pakistan was the first country to introduce TCV into its routine immunization schedule beginning in November 2019 [54]. It is anticipated that the prevention of typhoid fever, with the help of TCV, will likewise lead to reductions in TIP and its associated morbidity and mortality [21, 22, 32, 34, 37–40, 43]. Continued international support, especially from Gavi, will help to ensure that every child living in sub-Saharan Africa has access to this important vaccine.

In the USA and other high-resource countries, improvements in water, sanitation, and hygiene (WASH) programs have decreased the rates of typhoid fever and, in turn, TIP and other complications [2, 3, 5]. In addition, strengthening health systems, with improved access to care and surgery, and ensuring proper utilization by patients could also help to decrease the perforation to surgery interval, which is associated with increased surgical morbidity and mortality [55]. Unfortunately, WASH improvements and systems strengthening are difficult and expensive to implement in the short term in LMICs. Thus, vaccination becomes that much more important in this setting. Additional research is needed throughout sub-Saharan Africa, especially in rural areas, to highlight the burden of typhoid fever, and its complications, and to catalyze efforts to properly treat, and ideally prevent, this disease.

## Conclusion

Current estimates of mortality related to typhoid intestinal perforation among children in sub-Saharan Africa remain unacceptably high. While improvements in access to tertiary care are desirable, this is difficult and costly to implement in low-resource settings. Prevention of typhoid fever is therefore essential to help decrease mortality, with the goal of a comprehensive approach that utilizes vaccination, improvements in water, sanitation, and hygiene, and greater access to surgical care.

## Electronic supplementary material

Below is the link to the electronic supplementary material.

Supplementary file1 (DOCX 19 kb)
