# Supplementary material for: Morbidity and Mortality of Typhoid Intestinal Perforation Among Children in Sub-Saharan Africa 1995–2019: A Scoping Review
Source: World J Surg. 2020 May 19;44(9):2892–902. doi: 10.1007/s00268-020-05567-2 (PMC7236653; doi:10.1007/s00268-020-05567-2)
Supplement: Supplementary file 1 — Supplementary file1 (DOCX 19 kb) [file 268_2020_5567_MOESM1_ESM.docx]

**Online Resource 1**

PubMed Search Protocol

| PubMed Search Protocol | |
| --- | --- |
| Date of search | 07/31/2019 |
| Search String | ((“typhoid fever”[MeSH Terms] OR (“typhoid”[All Fields] AND “fever”[All Fields]) OR “typhoid fever”[All Fields] OR “typhoid”[All Fields]) OR “enteric fever”[All Fields] OR “typhoid fever”[All Fields]) AND (perforation[All Fields] OR (“peritonitis”[MeSH Terms] or “peritonitis”[All Fields]) OR (“ileum”[MeSH Terms] OR “ileum”[All Fields] OR “ileal”[All Fields])) AND (“1995/01/01”[PDAT]: “3000/12/31”[PDAT]) |
| Number of results | 347 |
